# The Amino Acid Transporter JhI-21 Coevolves with Glutamate Receptors, Impacts NMJ Physiology, and Influences Locomotor Activity in *Drosophila* Larvae

**DOI:** 10.1038/srep19692

**Published:** 2016-01-25

**Authors:** Anna B. Ziegler, Hrvoje Augustin, Nathan L. Clark, Martine Berthelot-Grosjean, Mégane M. Simonnet, Joern R. Steinert, Flore Geillon, Gérard Manière, David E. Featherstone, Yael Grosjean

**Affiliations:** 1 CNRS, UMR6265 Centre des Sciences du Goût et de l’Alimentation, F-21000 Dijon, France; 2 INRA, UMR1324 Centre des Sciences du Goût et de l’Alimentation, F-21000 Dijon, France; 3 Université de Bourgogne Franche-Comté, UMR Centre des Sciences du Goût et de l’Alimentation, F-21000 Dijon, France; 4 Biological Sciences, University of Illinois at Chicago, Illinois 60607, Chicago, USA; 5 Department of Computational and Systems Biology, University of Pittsburgh, Pittsburgh 15260, Pennsylvania, USA; 6 MRC Toxicology Unit, University of Leicester, LE1 9HN Leicester, UK

## Abstract

Changes in synaptic physiology underlie neuronal network plasticity and behavioral phenomena, which are adjusted during development. The *Drosophila* larval glutamatergic neuromuscular junction (NMJ) represents a powerful synaptic model to investigate factors impacting these processes. Amino acids such as glutamate have been shown to regulate *Drosophila* NMJ physiology by modulating the clustering of postsynaptic glutamate receptors and thereby regulating the strength of signal transmission from the motor neuron to the muscle cell. To identify amino acid transporters impacting glutmatergic signal transmission, we used Evolutionary Rate Covariation (ERC), a recently developed bioinformatic tool. Our screen identified ten proteins co-evolving with NMJ glutamate receptors. We selected one candidate transporter, the SLC7 (Solute Carrier) transporter family member JhI-21 (Juvenile hormone Inducible-21), which is expressed in *Drosophila* larval motor neurons. We show that JhI-21 suppresses postsynaptic muscle glutamate receptor abundance, and that JhI-21 expression in motor neurons regulates larval crawling behavior in a developmental stage-specific manner.

The glutamatergic *Drosophila melanogaster* larval neuromuscular junction (NMJ) is a powerful well-established model for the study of synaptic development and function. During the three larval stages the morphology of the NMJ changes dramatically^1^,^2^. From the hatching of a *Drosophila* larva up to the last larval instar, the muscle surface increases faster than the growth of the nerve terminals that innervate it. Despite this, the strength of these synapses is maintained at the same level^3^. This means that during larval development either the amount of released neurotransmitter or the receptivity of the muscle cell have to be adjusted. This could be achieved via a variety of mechanisms, including addition of new synapses to each junction, and changes in strength of individual synapses.

NMJ strength can also be tuned in previously unsuspected ways. In a previous study, for example, we identified a glial amino acid exchanger, Genderblind (GB), which is capable of tuning synaptic strength by regulating the amount of extracellular glutamate. This glutamate constitutively desensitizes ionotropic glutamate receptors (iGluRs), inhibits their clustering, and thereby suppresses synaptic transmission^4^,^5^. We also showed that the presynaptic neuron is capable of secreting non-vesicular glutamate through an unknown transporter to regulate NMJ strength by modulating iGluR clustering^6^.

In order to identify amino acid transporters that might regulate synaptic physiology during development, we used Evolutionary Rate Covariation (ERC). ERC is a recently established bioinformatic method that identifies functional relationships between proteins based on their evolutionary histories. The hypothesis of ERC is that functionally related proteins experience similar evolutionary selective pressures and hence have rates of evolution that correlate across species. ERC values are calculated by generating phylogenetic trees using full protein sequences and computing the correlation between the rates of change of two proteins across the branches of a phylogeny. The resulting values could range from 1 in case of positive correlation to -1 in case of negative correlation (Fig. 1a)^7^. ERC has previously been used to study proteins that are physically interacting or present in the same protein complex^8^,^9^,^10^,^11^,^12^. However, recent studies demonstrated that functionally related and coexpressed genes reveal positive and significant ERC values as well^7^. In this study, we screened for transporters showing evolutionary covariation with six glutamate receptor subunits. We hypothesized that the co-evolution of amino acid transporters and glutamate receptors might lead to the identification of unknown genes involved in glutamatergic signaling. We subsequently tested the functional relationship between those six GluRs and the transporters by taking advantage of the very well studied *Drosophila* larval NMJ physiology. Six GluRs have been shown to impact synaptic strength at this synapse: a metabotropic glutamate receptor subunit (mGluRA) expressed in motor neurons, and five iGluR subunits (GluRIIA, GluRIIB, GluRIIC, GluRIID, and GluRIIE) forming the ionotropic A- and B-type receptors expressed by the post-synaptic muscle cell^13^,^14^. We were particularly interested in transporters co-expressed with either mGluRA in motor-neurons or with iGluR subunits in muscle cells.

Using these criteria, we found a strong ERC value between these glutamate receptor subunits and the amino acid transporter JhI-21. Consistent with the idea that ERC predicts functional relationships, we found that JhI-21 negatively regulates iGluR clustering at NMJs and plays a role in locomotion control during late larval development. Investigating the reason for these effects, we discovered differential expression of JhI-21 in the central nervous system neurons and at the NMJ during larval feeding and wandering stages. Taken together, our results demonstrate how ERC can be used to find novel previously unsuspected roles for proteins, and reveal for the first time a role for JhI-21 in glutamatergic synapse function and behavior.

## Results

### JhI-21 is identified as a component of the glutamatergic signaling pathway by Evolutionary Rate Covariation (ERC)

**Figure 1:**
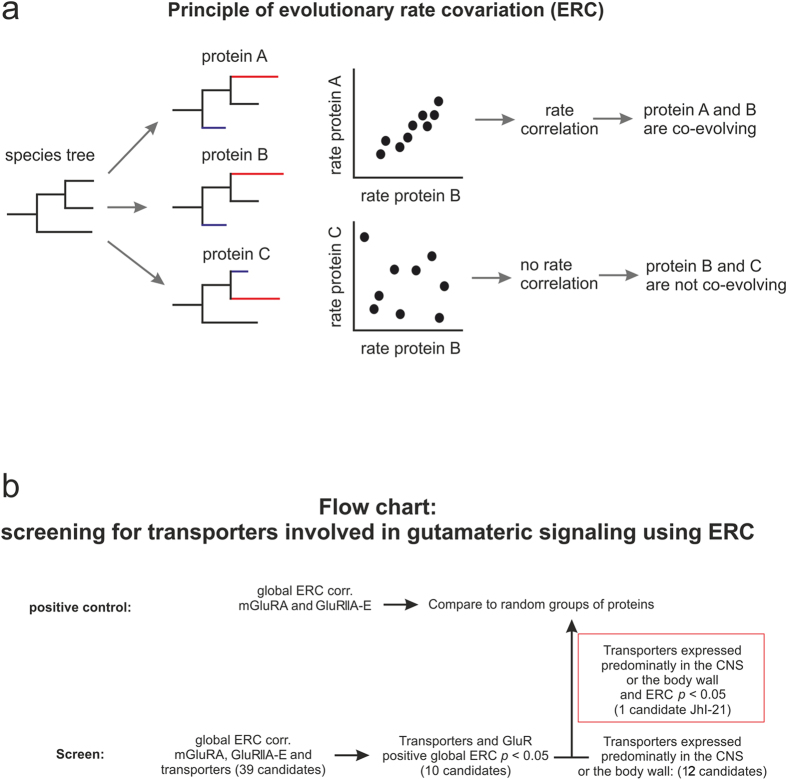
Evolutionary rate covariation. (**a)** The rates of evolution used in this study describe changes in protein sequences over time. To study if proteins are co-evolving, species trees were generated. In our study those trees were made by using homologue proteins of the following species: *D.melanogaster, D. simulans, D. sechellia, D. yakuba, D. erecta, D. ananassae, D. pseudoobscura, D. persimillis, D. wilistoni, D. mojavensis, D. virillis* and, *D. grimshavi*. The rates of a pair of proteins are plotted against each other and a factor is calculated describing the correlation of these rates. The rates of co-evolving proteins (protein A and B) are positive while proteins, which are not co-evolving (protein B and C) have correlation coefficients close to zero or negative. (**b**) As a positive control we calculated the global ERC value for the six glutamate receptors (mGluRA and iGluRA-E), which are known to act together at the *Drosophila* NMJ. For statistical analysis a mean correlation was calculated and compared to the mean correlation of random sets of six proteins. Next ERC values were calculated between the 39 transporter candidates and the GluRs mentioned above. Ten transporter candidates were showing robust ERC values (*p < *0.05) with each glutamate receptor. Next we compared the expression pattern of the six GluRs with the expression pattern of the putative transporters. mGluR is predominantly expressed in the CNS, iGluRs in the carcass. Twelve putative transporters were showing their highest level of expression in either the CNS or the body wall ( www.flybase.org), JhI-21 showed robust ERC with GluRs and was expressed highest in the same tissue than GluRs, and was chosen for further investigation. For statistical analysis the mean correlation of JhI-21 and the six GluRs was compared to a random groups of 7 proteins.

Six glutamate receptor subunits (mGluRA, GluRIIA-E) are known to be key members for signal transmission at the glutamatergic 3^rd^-instar larval NMJ. We identified their orthologs in 12 *Drosophila* species and calculated their ERC values (Fig. 1b). The six glutamate receptors showed overall positive scores indicating robust rate covariation. The mean ERC value between all possible pairs of those 6 proteins is 0.556 and is strongly significant (*p* = 0.00021, permutation test; Table 1). Positive ERC values are typically found for proteins acting as subunits in the same complex, as in the case for the ionotropic glutamate receptor subunits GluRIIA-E. Glutamate receptors in larval NMJs localize to postsynaptic densities on the surface of the muscle cell and always contain GluRIIC, GluRIID, and GluRIIE subunits. In addition, “A-Type” receptors contain GluRIIA while “B-Type” receptors contain GluRIIB. In contrast, the metabotropic mGluRA receptor subunit is expressed in the innervating motor neuron, and acts in a glutamatergic feedback loop regulating the amount of transmitter released^15^. Even though metabotropic and ionotropic glutamate receptors are not co-localized within the same cell at the NMJ of 3^rd^-instar larvae, they do act in the same intercellular signaling pathway and therefore the ERC values between the two different types of receptors are very high. These conditions demonstrate that ERC can detect proteins that function together despite being expressed in different cells. This finding enabled us to use this technique to screen for previously unrecognized candidate amino acid transporters associated with glutamatergic signal transmission.

**Figure 2:**
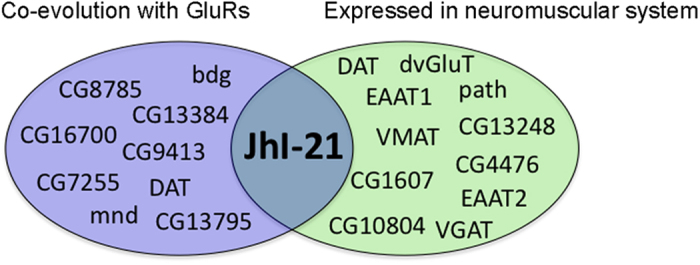
Candidate amino acid transporters involved in glutamatergic transmission. The *Drosophila* genome encodes 39 genes showing homology to amino acid transporters^4^,^44^. 10 of those were predicted to be co-evolving with GluRs by usage of ERC (blue) (ERC-values >0.23 and *p* < 0.05). Out of those 39 putative transporter genes 11 show the highest level of expression in the same tissue as either mGluRA (highest expression in the CNS) or iGluRs (highest expression in the body wall) (green). We picked one candidate gene, JhI-21, for further analysis.

In total, we first screened 39 confirmed and putative amino acid transporter homologs^16^,^17^ for rate covariation with the six glutamate receptors mentioned above (Table 2 and Supplemental Table1). Ten putative transporters showed positive and significant (*p* < 0.05) ERC values with the six glutamate receptor subunits (Table 2, Fig. 2). To prove the functional relationship between these GluRs and our positive hits we decided to focus next on the glutamatergic larval NMJ. The cell bodies of motor-neurons expressing mGluRA are located in the ventral nerve cord of the larval CNS. Those neurons project their axons towards the body wall where they contact muscle cells, which express iGluRs. Therefore, in parallel we used a second screening procedure to determine which transporters are most highly expressed in either the CNS (where motor neuron cell bodies are housed) or the body wall (location of NMJs) (Tables 1 and 2). We found eleven putative transporter genes (Table 2, Fig. 2). We selected one significant hit from our ERC analysis, JhI-21, which also matched very well our second criterion concerning the expression pattern in the neuromuscular system (Fig. 2).

The single ERC values of JhI-21 compared to the different glutamatergic receptors range from 0.298 to 0.59 (Supplemental Table 1). The global ERC value for JhI-21 and the six glutamate receptor subunits that we examined is 0.3788 (*p* = 0.0019, permutation test) (Table 2). This very high co-evolutionary statistic suggested that JhI-21 and the glutamate receptor subunits co-evolved and therefore may be functionally related.

JhI-21 was initially discovered in a screen for juvenile hormone (JH) inducible genes^18^,^19^. It was classified as a SLC7A5-11 family member based on sequence analysis^4^, and amino acid uptake into cultured cells^20^. The *Drosophila* genome encodes four more putative SLC7A5-11 paralogs: Genderblind (GB), Minidiscs (Mnd), CG9413 and CG1607^4^,^20^. GB controls extracellular glutamate levels, which in turn regulates the number of iGluRs in the glutamatergic NMJ^4^,^21^. This unexpected function -regulation of iGluRs in synapses- was recently shown to be also conserved in mice^22^.

We therefore turned our attention to testing explicitly whether JhI-21 and iGluRs in the *Drosophila* are functionally related, as predicted by ERC.

### JhI-21 is expressed in motor neurons at the glutamatergic NMJ of 3^rd^ instar larvae

**Figure 3:**
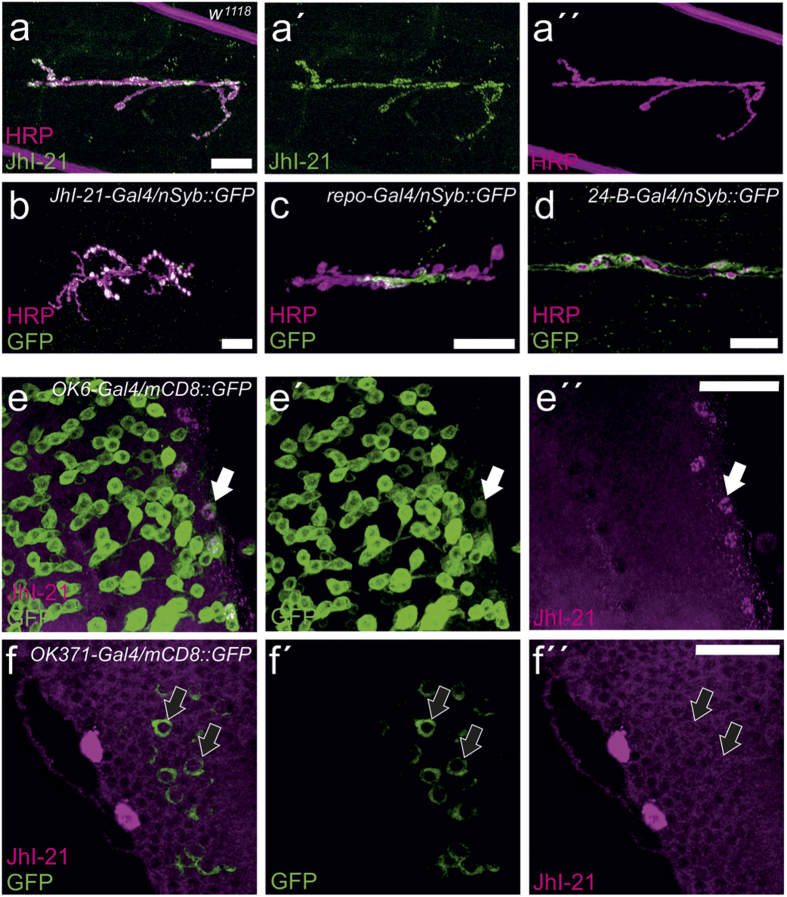
JhI-21 is expressed in presynaptic motor nerve terminals. (**a**) Confocal image of third-instar larval NMJ formed on ventral longitudinal muscles 6 and 7 marked by anti-HRP (magenta) co-stained with antibodies against JhI-21 (green). (**b–d**), Representative confocal projections of third-instar larval NMJs formed on ventral longitudinal muscles 6 and 7, stained with antibodies against HRP (magenta) and against GFP (green). Only *JhI-21*-Gal4 driven transgenic synapse-tethered GFP (nSyb::GFP) expression co-localizes with anti-HRP labeling (B). Glial (*repo*)-Gal4 or muscle (24-B)-Gal4 driven nSyb::GFP does not show co-localization with anti-HRP labeled motor-neurons. (**e**,**f**) Ventral nerve cord (VNC) of third instar larva. (E) Cell bodies of motor-neurons marked by OK6-Gal4 driving the expression of membrane bound mCD8GFP (green), and are co-labeled by anti-JhI-21 (magenta). (F) Cell bodies of glutamatergic neurons marked by OK371-Gal4 driving the expression of membrane bound mCD8GFP (green), and are co-labeled by anti-JhI-21 (magenta). Two arrowheads are pointing two examples of cells expressing both the GFP marker and JhI-21. Scale Bar NMJ = 20 mm. Scale Bar VNC = 40 μm.

We first investigated whether JhI-21 is expressed at the larval neuromuscular junction close to the glutamate receptor subunits with which ERC revealed it to coevolve. The *Drosophila* larval NMJ is a tripartite synapse containing the presynaptic motor neuron, the postsynaptic muscle cell, and adjacent glia. All three cell-types are known to be involved in regulating synaptic development and physiology^23^. First, we designed a polyclonal anti-JhI-21 antibody and confirmed its specificity in JhI-21 null mutant embryos (Figure S1). As shown in Fig. 3a, we could detect the anti-JhI-21 labeling at the NMJ. This anti-JhI-21 signal co-localized with the anti-HRP, which marks presynaptic motor terminals. These data therefore strongly suggest that JhI-21 is expressed in motor neuron terminals.

However, the close proximity of different cell types (neurons, muscle, or glia) at the NMJ makes it difficult to conclude cell-type expression based on light microscopy alone. To address this problem, we generated transgenic flies in which Gal4 is expressed under the control of a putative *JhI-21* regulatory region, and then examined the expression pattern of UAS-nSyb::GFP using the Gal4/UAS system^24^ (Fig. 3b). Synaptobrevin (Syb) is a synaptic terminal protein, and therefore nSyb::GFP will be enriched at synaptic endings when expressed in neurons^25^. Consistent with the conclusion that JhI-21 is expressed in motor neurons, nSyb::GFP expressed under control of *JhI-21*-Gal4 was localized to synaptic terminals. To ensure that nSyb::GFP did not localize similarly when expressed in glia or muscles, we also expressed nSyb::GFP under the control of well-characterized glial and muscle cell Gal4 drivers (repo-Gal4 and 24B-Gal4, respectively; Fig. 3c,d). As expected, the nSyb::GFP fluorescence pattern in these cases was drastically different from when nSyb::GFP is expressed in motor neurons. We therefore conclude that JhI-21 is normally expressed in motor neurons and (based on antibody staining) at least in part localized to motor neuron terminals.

To determine whether JhI-21 was expressed in other parts of the motor neurons, we examined the expression of JhI-21 protein in the ventral nerve cord (VNC), and compared it to the location of glutamatergic motor neurons cell bodies marked using the OK6-Gal4, which is expressed in motor neurons, or OK371-Gal4, which is expressed in glutamatergic neurons, using membrane-bound GFP (mCD8::GFP) as a reporter transgene. As shown in Fig. 3e, anti-JhI-21 labeling was detected in cell bodies at the VNC. While most neurons appear to express low levels of JhI-21, some neurons identified as a subset of glutamatergic motor neurons by the OK6-Gal4 or OK371-Gal4 driven expression of mCD8::GFP, express JhI-21 at a higher level (solid arrow in Fig. 3e; white bordered arrow in Fig. 3f). According to the results obtained with our anti-JhI-21 antibody and our *JhI-21*-Gal4 transgene, JhI-21 seems to be expressed in neurons of 3^rd^-instar larvae, with highest expression in motor neurons (Fig. 3, Figure S2). However, our *JhI-21*-Gal4 transgene recapitulates just partially the endogenous expression of JhI-21 expression in the brain (Figure S2), suggesting that JhI-21 expression in motor neurons may be variable or actively regulated.

### Loss of JhI-21 expression increases iGluR clustering in NMJs

**Figure 4:**
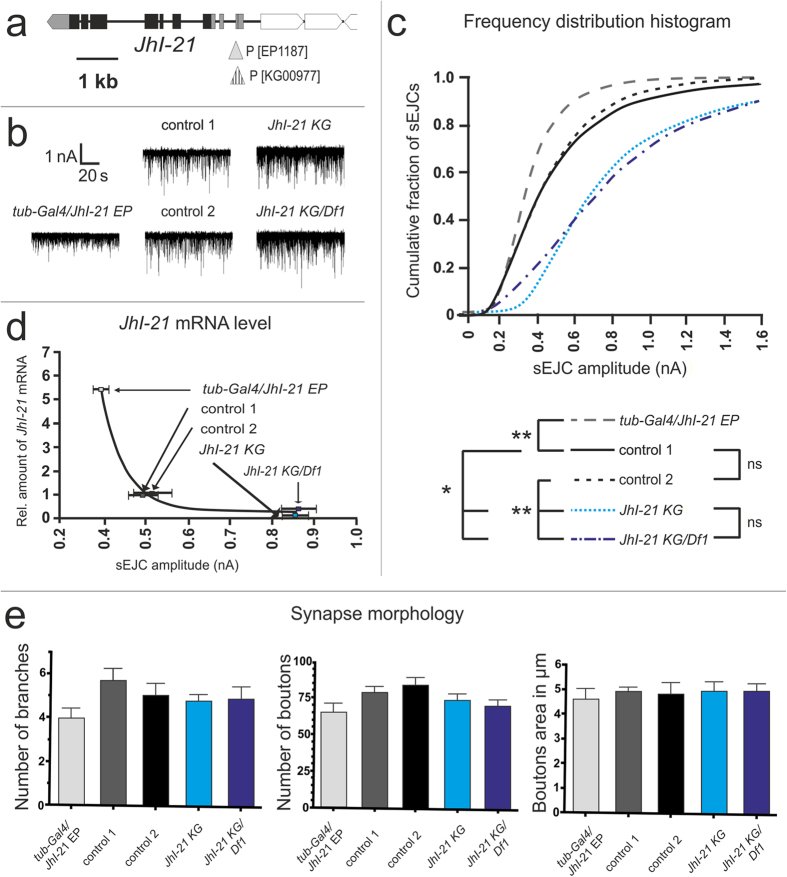
The electrophysiological response at the muscle 6/7 NMJ is controlled by JhI-21. (**a**) Schematic representation of the *JhI-21* genomic locus. Exons are indicated by boxes, translated exons of the *JhI-21* gene by black boxes, 5´and 3´untranslated regions of the *JhI-21* gene as gray boxes. Triangles represent the inserting region of P-elements used to generate hypo-(stripped triangle) or hypermorph (grey triangle) *JhI-21* alleles. (**b**) Representative traces for two-electrode voltage clamp experiments from the larval muscle 6 (LIII). As wildtype we used *w*^*1118*^ (control 1) and a clean excision of P(KG00977) (control 2). (**c**) Relative cumulative frequency histogram of sEJC (“mini”) amplitudes from different genotypes in third-instar *Drosophila* larvae. Rightward shift (*JhI-21 KG* and *JhI-21 KG/Df1*, blue) indicates increase in the abundance of current-conducting postsynaptic receptors, i.e larger synaptic currents. Note the shift to the left (i.e. decreased receptor number) in JhI-21 overexpression mutants (gray) N = 6-10 animals, 800-3.400 events measured. Kolmogorov-Smirnov test was used to compare the cumulative distributions two by two (*P < 0.05; **P < 0.01). (**d**) Negative correlation between the *JhI-21* mRNA levels and the number of sEJC amplitudes at the NMJ. (**e**) Motor neurons were stained using anti-HRP and confocal images were taken at the 6/7 NMJ. Neither *JhI-21 KG* nor *JhI-21 KG/Df1* showed alterations of synaptic morphology in terms of NMJ branches, bouton number, or bouton area using a Kruskal-Wallis test. Error bars represent SEM in d and e.

The expression pattern of JhI-21, along with its co-evolution with glutamate receptors, raised the possibility that JhI-21 regulates NMJ physiology. To test this hypothesis, we performed two-electrode voltage-clamp electrophysiology to measure spontaneous excitatory junction currents (sEJCs) in control and *JhI-21* mutant larval NMJs (muscle 6, segment A3). We used the hypomorphic *JhI-21* allele P{SUPor-P}*JhI-21*[KG00977], (hereafter referred to as ‘*JhI-21 KG’),* which was generated by the BDGP Gene Disruption Project and carries a P{SUPor-P} transposable element in the first exon of the *JhI-21* gene^26^. The *JhI-21 KG* allele was used homozygous or in combination with a deficiency (*Df1* is *Df(2L)esc-P3-0*), in which the *JhI-21* gene is deleted^27^. Another strain, P*JhI-21*[EP1187] (*JhI-21 EP*), contains UAS-binding sites and was used to over-express JhI-21^28^. As controls, we used *w*^*1118*^ (control 1) and P*82 (control 2). P*82 is a clean excision allele of the *JhI-21* P-element [*KG00977*] (Fig. 4a)^29^. As shown in Fig. 4d, sEJC amplitudes are strongly correlated with *JhI-21* expression as measured by q-PCR. sEJC amplitude distributions measured in the two control strains are nearly indistinguishable from each other (P > 0.05, n.s., Kolmogorov-Smirnov test). The *JhI-21* hypomorphs, however, display significantly larger spontaneous postsynaptic currents (P < 0.05). In contrast, larvae overexpressing *JhI-21* show smaller postsynaptic currents (P < 0.01 compared to control 1; Fig. 4b,c; Figure S3).

sEJC frequencies were not significantly different between genotypes (control 1 = 2.6 ± 0.5 Hz; control 2 = 2.3 ± 0.3 Hz; *JhI-21 KG* = 1.9 ± 0.2 Hz; *JhI-21 KG/Df1* = 2.5 ± 0.3 Hz; *tub*-Gal4/*JhI-21* EP = 1.7 ± 0.3 Hz; n.s.) ruling out the possibility of changes in presynaptic release.

As expected based on changes in sEJC size, overexpression of JhI-21 in the *JhI-21 EP* strain led to a reduction in evoked excitatory junction currents (eEJCs; Figure S4). This suggests that increased JhI-21 leads to less muscle excitation. However, we observed no significant differences between the controls and loss-of-function mutant genotypes (Figure S4).

The number of NMJ branches, number, and bouton area were not statistically different in between controls and *JhI-21* mutant alleles (Fig. 4e).

The dramatic changes in sEJC amplitude without apparent changes in NMJ morphology or frequency of neurotransmitter release suggest that *JhI-21* mutant NMJs might cause alterations in the number of postsynaptic glutamate receptors. To test this, we measured postsynaptic glutamate receptor protein abundance immunohistochemically.

*Drosophila* muscle cells express two different subtypes of heterotetrameric iGluRs, called ‘A-type’ and ‘B-type’, which can be distinguished immunocytochemically using antibodies against the subunits unique to each receptor type: GluRIIA, and GluRIIB^30^,^31^,^32^,^33^.

**Figure 5:**
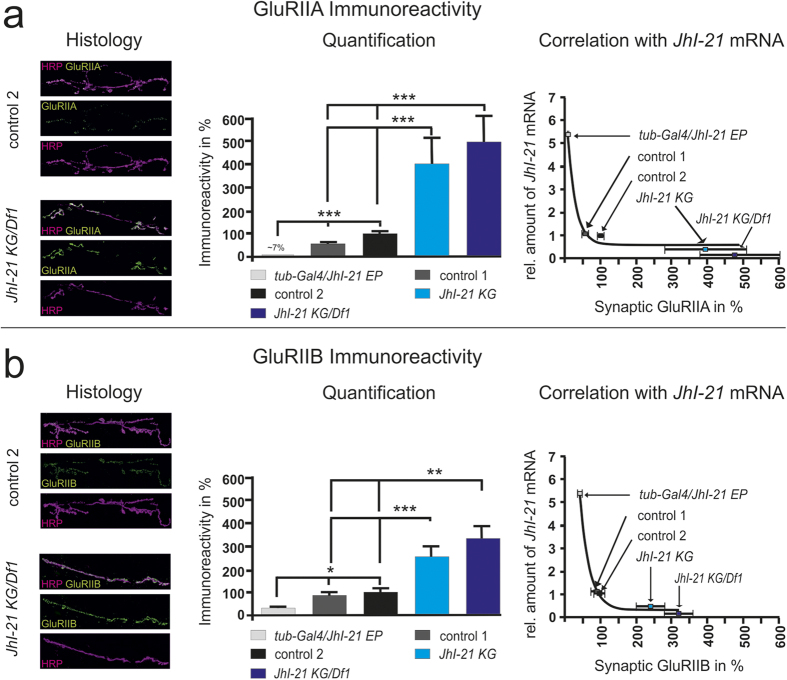
Postsynaptic glutamate receptor immunoreactivity is inversely proportional to *JhI-21* expression levels. Genotypes used are the same than the ones shown in Fig. 2. (**a**) *Left*, representative confocal images showing the accumulation of GluRIIA receptor subunits at the NMJ in *JhI-21* hypomorphs (*JhI-21 KG*/*Df1*) compared to a control genotype control 2; *middle*, quantification of the NMJ GluRIIA and GluRIIB abundance in various genotypes; *right*, negative correlation between the *JhI-21* mRNA levels and the abundance of type-A glutamate receptor subunits at the NMJ. (**b**) Immunohistochemistry of type B glutamate receptor subunits (GluRIIB) shows similar (negative) correlation with *JhI-21* transcript levels (N = 4–13 animals). Error bars represent SEM; statistical test: Kruskal-Wallis test. (*p < 0.05; **p < 0.01; ***p < 0.001).

**Figure 6:**
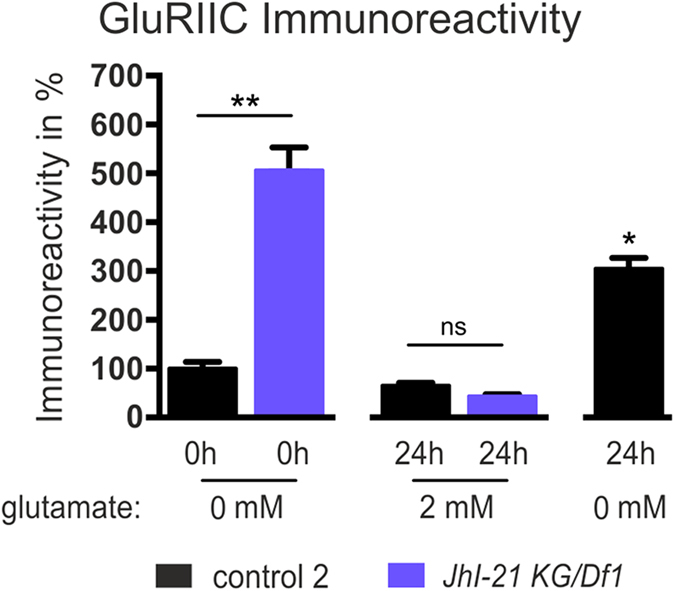
Postsynaptic GluRIIC immunoreactivity is dependent on *JhI-21* expression and ambient glutamate. Bar graphs showing postsynaptic GluRIIC abundance in a control genotype (precise excision of *JhI-21 P*[*KG00977*]) and a JhI-21 hypomorph allele *JhI-21 KG/Df1*, and showing postsynaptic reduced GluRIIC abundance in *JhI-21 KG/Df1* mutants incubated with 2 mM ambient glutamate or without for 0h or 24 hours. (N = 3-6 animals per genotype and condition). *p < 0.05 (control compared to the other incubation times 2 by 2); **p < 0.01; ns: not significant (Mann-Whitney tests). Error bars represent SEM.

Using anti-GluRIIA and anti-GluRIIB antibodies, we compared the immunoreactivity for both A- and B-type receptors in wild-type controls and in the *JhI-21* alleles. The strongest *JhI-21* hypomorph allele (*JhI-21 KG/Df1*) causes a ~5-fold increase in the postsynaptic receptor abundance for A-type receptors and a ~3.5-fold increase in B-type receptors compared to control. In contrast, overexpression of JhI-21 (*tub*-Gal4/*JhI-21* EP) caused a significant decrease in A- and B-type receptors compared to control genotypes at the NMJ (Fig. 5). We obtained the same effect when looking at the GluRIIC subunit, which is shared by A- and B-type receptors (Fig. 6). Overall, as observed for sEJC amplitudes, the iGluR immunoreactivity was negatively correlated to the amount of larval *JhI-21* mRNA detected by q-PCR (Fig. 5). Taken together, these results show that JhI-21 negatively regulates glutamatergic transmission and the abundance of iGluR protein at the larval NMJ.

We next wondered if the action of JhI-21 on iGluR clustering could be through the action of glutamate, based on previous work showing that the related transporter GB controls iGluR abundance via regulation of extracellular glutamate^4^,^5^. Consistent with this idea, we could fully rescue the phenotype of our strongest mutant by bathing *JhI-21 KG/Df1* NMJs with 2 mM glutamate during 24 h when measuring GluRIIC staining (Fig. 6). Unfortunately our intense efforts to test if JhI-21 could transport (or not) glutamate, by glutamate quantification in the hemolymph or by using the S2 cell model system, failed to give conclusive results (Figure S7).

### JhI-21 regulates locomotor behavior

Our results reveal JhI-21 as an unexpected regulator of NMJ iGuR abundance and spontaneous synaptic transmission strength. But what role does this novel form of regulation play ? To test whether changes in JhI-21 expression at the NMJ and/or in the CNS could have behavioral consequences we measured speed and meandering (turning rate) at two different physiological stages of 3^rd^-instar larvae: feeding and wandering (shortly before pupation).

**Figure 7:**
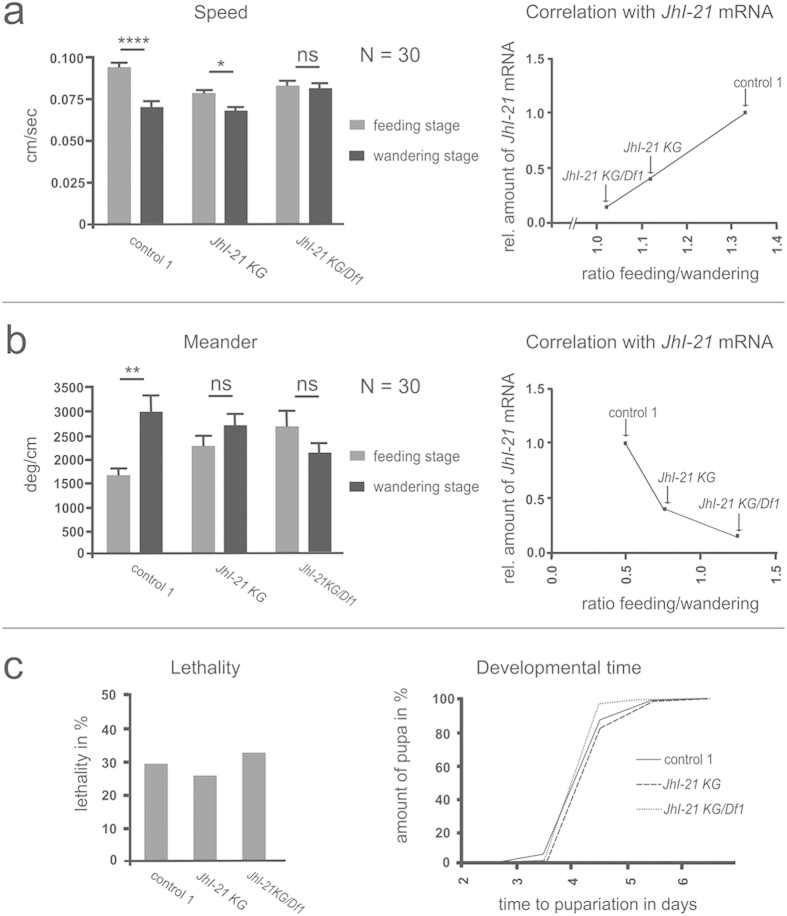
*JhI-21* mutants lack age-dependent shift in locomotor behaviors. (**a,b**) Parameters of locomotion were analyzed in wildtype larvae (control 1, *w*^*1118*^) and *JhI-21* mutants (*JhI-21 KG* and *JhI-21 KG*/*Df1*). (**a)** right, Speed is significantly decreased in wandering wildtype larvae compared to feeding individuals. ****p < 0.0001. This difference is still present, although reduced in *JhI-21 KG* mutants. *p < 0.05. Difference in speed between feeding and wandering larvae is not present in *JhI-21 KG*/*Df1* mutants. Left, the ratio of average speed of feeding larvae/average speed of wandering larvae correlates with *JhI-21* mRNA levels. (**b)** right, the meander describes amount of turnings in degree per cm and was used to characterize the turning behavior. 3^rd^ instar wildtype larvae increase their average turning in wandering stage. **p < 0.01. Neither *JhI-21 KG* nor *JhI-21 KG*/*Df1* mutants show a significant change in turning behavior between both 3^rd^-instar larval stages. Left, the ratio of average meander of feeding larvae/average speed of wandering larvae shows negative correlation with *JhI-21* mRNA levels. (**c**) JhI-21 mutants show no difference in lethality or developmental time as compared to the control genotype. Statistical test in (**a,b**): 2-Way-ANOVA, N = 30, Error bars represent SEM. Statistical test in c Mantel-Cox test: N = 131-334 per genotype.

In the absence of food, wildtype feeding-stage larvae moved significantly faster than wildtype wandering-stage larvae (0.093 ± 0.003 cm/sec, and 0.070 ± 0.003 cm/sec respectively; *p* < 0.0001). Also, feeding-stage larvae had a lower turning rate compared to the wandering-stage larvae (1651 ± 151 deg/cm for feeding-stage larvae compared to 2918 ± 335 deg/cm for wandering ones; *p* < 0.01; Fig. 7a,b).

To test whether JhI-21 regulates locomotor behavior, we used the hypomorphic *JhI-21 KG* allele. Wandering homozygous *JhI-21 KG* hypomorphs still showed a significant decrease in speed (from 0.078 ± 0.003 cm/sec in feeding stage to 0.066 ± 0.003 cm/sec in wandering stage; *p* < 0.05), but no change in meandering (2263 ± 210 deg/cm in feeding stage versus 2677 ± 269 deg/cm in wandering stage; n.s.). In the strongest viable mutants, *JhI-21 KG/Df1* hypomorphs, neither speed (0.083 ± 0.003 cm/sec in feeding stage and 0.081 ± 0.003 cm/sec in wandering stage) nor meandering (2685 ± 331 deg/cm in feeding stage and 2126 ± 195 deg/cm in wandering stage) differed between feeding and wandering larvae (Fig. 7a,b; Figure S5). Therefore, *JhI-21* mutants do not exhibit normal differences between feeding and wandering larvae in locomotor characteristics (speed and meandering) that are displayed by control animals.

To confirm the association between *JhI-21* expression level and locomotor behavior, we divided within each genotype the mean value scored for feeding animals by the mean value scored for wandering animals, and plotted this ratio against the average *JhI-21* mRNA levels. The ratio for speed negatively correlates with *JhI-21* mRNA levels, while the ratio for meandering positively correlates with the expression levels of *JhI-21* mRNA (Fig. 7a,b).

We also looked at other locomotor phenotypes such as the number of stops and go, and the peristaltic waves along the length of the larval body axis. No difference between feeding and wandering larvae were found (Figure S6). Since all genotypes tested in this assay showed also no difference in developmental time or lethality (Fig. 7c), *JhI-21* expression at the NMJ and/or in the CNS seems to specifically regulate locomotor behavior shifts (speed and meandering) that normally occur in late 3^rd^-instar larvae.

### Synapse physiology differs in feeding and wandering 3^rd^-instar larvae

**Figure 8:**
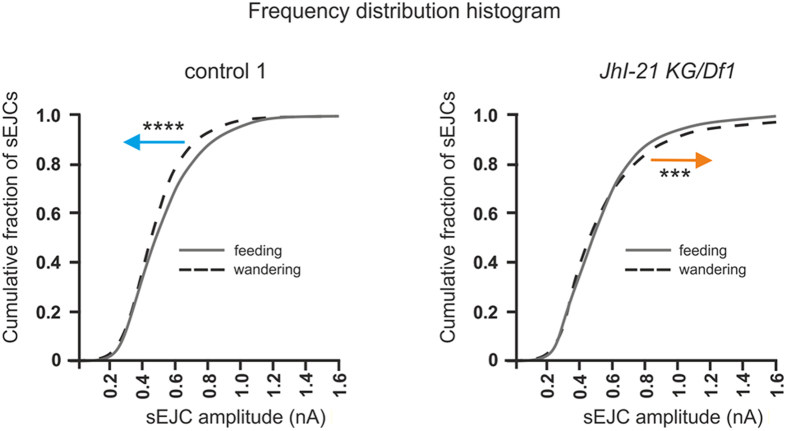
The age-dependent shift in electrophysiological response at the muscle 6/7 NMJ is controlled by JhI-21. Cumulative frequency histograms of sEJC (“mini”) amplitudes from third-instar feeding and wandering *Drosophila* larvae. Leftward shift in wandering control animals indicates an age-dependent decrease in the abundance of current-conducting postsynaptic glutamate receptors, i.e smaler synaptic currents. This shift is inverted to rightward in the *JhI-21 KG/Df1* allele. N = 4–5 animals (representing 10–17 NMJs), 2400–3600 events measured per phenotype; Statistical test: Kolmogorov-Smirnov test (***P < 0.001, ****P < 0.0001).

If JhI-21 regulates NMJ physiology, and this regulation affects feeding and wandering behavior, then there should be differences in NMJ physiology between feeding and wandering larvae. To test this, we compared sEJC amplitude distributions in feeding and wandering animals. In our control genotype we observed overall smaller amplitudes of sEJCs when larvae were in the wandering stage compared to feeding stage larvae (*p*<0.0001). This change could be explained by JhI-21-mediated inhibition of iGluR clustering at the postsynaptic muscle cell during the wandering stage (Fig. 4). To test this hypothesis explicitly, we compared sEJCs in our strongest JhI-21 hypomorph (*JhI-21 KG/Df1*). The JhI-21 hypomorphs exhibit overall larger spontaneous miniature postsynaptic currents in wandering stage compared to feeding 3^rd^-instar larvae, consistent with the hypothesis (*p*<0.001; Fig. 8).

### JhI-21 is differentially expressed in feeding and wandering 3^rd^-instar larvae

**Figure 9:**
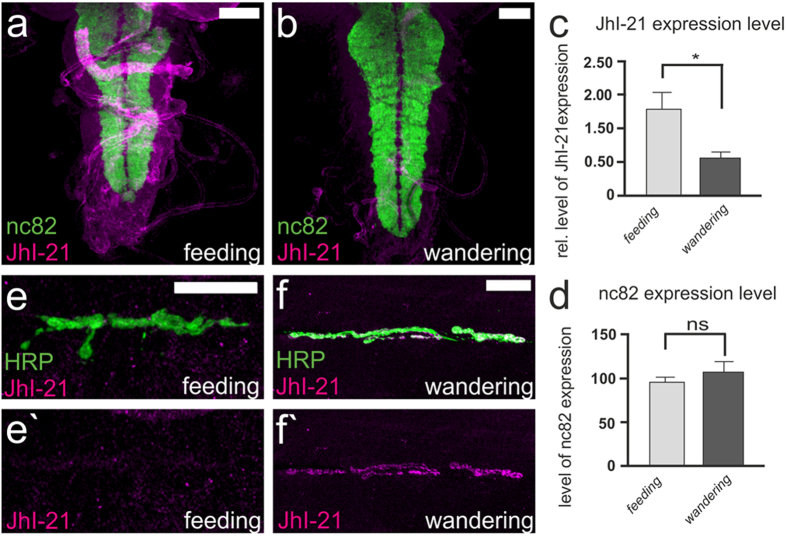
JhI-21 is differentially expressed in the nervous system of feeding and wandering 3^rd^-instar control larvae. (**a**,**b)** Confocal projection of *w*^*1118*^ 3^rd^-instar larval VNC stained with anti-JhI-21 (magenta) and NC82 antibody (green). (**c,d)** Quantification of signal intensity reveals higher expression of JhI-21 in the ventral nerve cord of feeding larvae compared to wandering ones (**p < 0.01, N = 12, Mann Whitney test). Intensity of nc82 does not change between these larval stages (N = 12, unpaired t-test). (**e,f)** Confocal images of third-instar larval (LIII) NMJs of muscles 6 and 7, stained with antibodies against HRP (stains all neuronal membrane, green) and JhI-21 (magenta). At the NMJ anti-JhI-21 labeling is detectable at wandering stage, not at feeding stage. Scale Bar (brains) =40 mm. Scale Bar (NMJs) =20 mm. Error bars represent SEM in c and d.

How does JhI-21 regulate feeding and wandering behavior? One possibility is that JhI-21 expression at the NMJ, and thus the strength of NMJ regulation, differs between feeding and wandering stages. To test this we stained the nervous system of feeding and wandering 3^rd^ instar larvae with the anti-JhI-21 antibody and analyzed the expression levels. In the VNC, where the cell bodies of motor neurons are located, expression of JhI-21 is significantly higher in feeding animals than in wandering ones (feeding stage 1.275 ± 0.266, wandering stage 0.531 ± 0.117; *p < *0.05) (Fig. 9a–c). The anti-Bruchpilot antibody nc82 labels the neuropil^34^ and was used as an internal control for this experiment. As shown in Fig. 9d, the signal obtained with nc82 was constant between feeding-stage (94 ± 6) and wandering-stage larvae (107 ± 10). In contrast JhI-21 staining could never be detected at the NMJ of feeding-stage animals, but wandering stage animals showed strong anti-JhI-21 labeling in the NMJ (Fig. 9e,f). Thus, JhI-21 subcellular localization appears to shift between feeding and wandering stages. During feeding stage, JhI-21 is predominantly in the cell bodies within the VNC and not in the motor terminals. During wandering stage, JhI-21 is strongly localized in the motor terminals at the NMJ, and less abundant in the cell bodies within the VNC.

## Discussion

### Evolutionary rate co-variation identified JhI-21 as a member of glutamatergic signaling

In this study we used the “covariation of protein evolutionary rates” to screen for proteins that might play unsuspected roles in glutamatergic synapse physiology. It has been previously demonstrated that ERC signatures provide a powerful method to reveal functionally related proteins or proteins acting in the same complex^8^,^35^,^36^. As expected, physically interacting ionotropic glutamate receptor (iGluR) subunits GluRIIA, GluRIIB, GluRIIC, GluRIID, and GluRIIE showed overall positive ERC values. Even if the metabotropic glutamate receptor mGluRA is neither physically interacting nor co-expressed in the same cell than iGluR subunits, it also showed positive ERC values when compared to iGluR subunits, likely due to its action in the same neurophysiological pathway. We therefore considered whether the function of two proteins in the same synapse can be sufficient to gain positive ERC values even though both the proteins are not expressed in the same cell.

We next used ERC to determine whether any of 39 putative amino acid or biogenic amine transporters genes were co-evolving with GluRs, and got 10 significant hits. Based on ERC scores and tissue expression, we selected the amino acid antiporter JhI-21 for further analysis. Specifically, we sought to determine whether JhI-21 did indeed function in the glutamatergic signaling pathway as suggested by ERC.

Using two independent strategies we showed that JhI-21 is localized in glutamatergic motor neurons in 3^rd^-instar larvae. JhI-21 showed the strongest correlation in terms of co-evolution with mGluRA, which is also expressed in motor neurons^15^. Although co-expressed proteins tend to have higher ERC values in general^7^, we want to highlight that JhI-21 also showed positive ERC score with the post-synaptically expressed iGluRs, indicating a previously unsuspected but important role for JhI-21 in adjusting the strength of glutamatergic neuromuscular transmission.

Interestingly, other significant hits are not even expressed in the CNS or the body wall. For example CG8785 is expressed only in the digestive system and malpighian tubules. Both tissues also contain iGluRs. What might be the role of iGluRs in the digestive and *Drosophila* renal system and what could be the link to the co-evolution with CG8785 is an interesting subject for further studies.

### Possible direct action of JhI-21 in adjusting synaptic strength at the larval NMJ

The fact that JhI-21 is expressed at the glutamatergic synapse allowed us to further elucidate the functioning of the NMJ in *Drosophila*. Specifically, we found that JhI-21 expression in motor neurons leads to inhibition of synaptic transmission by reducing the clustering abundance of iGluRs in neuromuscular junctions of late stage 3^rd^-instar larvae. This could be linked to the extracellular glutamate concentration since additional application of glutamate can compensate a lack of JhI-21 activity in *JhI-21* hypomorph mutants when measuring the amount of iGluR expression at the NMJ (Fig. 6). It is therefore reasonable to ask whether JhI-21 has a direct impact on extracellular glutamate levels at the NMJ.

**Figure 10:**
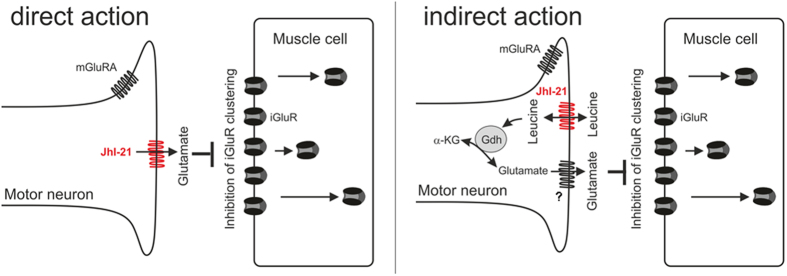
JhI-21 co-evolved with glutamate receptors, and regulates strength of glutamatergic signaling at the larval NMJ. JhI-21 is expressed in motor neurons together with mGluRA, and regulates synaptic strength by inhibiting the clustering of iGluRs at the postsynaptic muscle cell specifically during the wandering stage in third-instar larvae. The schematic representation illustrates two possible functions of JhI-21 on iGluR clustering in wandering larvae. On the left is shown the direct export of glutamate close to the synapse, which inhibits iGluR clustering. On the right, an indirect action of JhI-21 is proposed through the transport of leucine. Leucine acts on the activity of the Glutamate dehydrogenase (Gdh), which can reciprocally catalyze the production of a-ketoglutarate (a-KG) to glutamate within the motoneuron. Glutamate then could be released to the hemolymph surrounding the NMJ via an unidentified transporter, and act on iGluR clustering.

Indeed JhI-21 was classified as a SLC7A5-11 family member based on sequence analysis^4^. These transporters can be found in many species across the animal kingdom and other members of this transporter family have been proven to regulate glutamatergic signaling directly by adjusting extracellular glutamate levels in mammals and *Drosophila*^4^,^20^. This extracellular glutamate is mostly independent of synaptic vesicular release^6^,^37^,^38^, and is partly attributable to glial expressed SLC7A11 transporters^4^,^21^,^39^. Those transporters in mammals act as hererodimers consisting of a ‘light chain’ SLC7A5-11 core catalytic subunit in combination with a ‘heavy chain’ subunit (such as 4F2hc protein) that is required for trafficking to the plasma membrane^40^,^41^,^42^,^43^. GB, which is the homologue of JhI-21, previously revealed to be expressed in a particular subset of glia at the larval NMJ, was the first *Drosophila* SLC7A5-11 member shown to directly impact the physiology of this glutamatergic synapse by exporting glutamate into the hemolymph^4^,^44^. This led us speculate that JhI-21 might also export glutamate directly. Nevertheless, when compared to GB, JhI-21 does not affect the overall hemolymph glutamate concentration^29^. Presumably, JhI-21 modulates postsynaptic iGluR levels via some other mechanism, or regulates glutamate only very locally near the synapse, in contrast to GB, which controls glutamate levels more globally (Fig. 10). Using JhI-21 expressing S2 cell we could not demonstrate that this transporter might directly or not transport glutamate (Figure S7), although we found that incubating larval NMJs in glutamate was able to rescue the JhI-21 phenotype (Fig. 6).

We also considered whether JhI-21 might be involved in loading glutamate into neurotransmitter vesicles and might thereby have a direct impact in glutamatergic transmission. No SLC7A5-11 family member is known to be a component of synaptic vesicle membrane. Also it has been shown that at the larval NMJ the vesicular glutamate transporter (VGlut, belonging to the SCL17 family) is necessary and sufficient to fill vesicles with glutamate. Absence of VGlut leads to empty vesicles at the NMJ^45^. This indicates that there is no other transporter involved in the filing of glutamate into these neurotransmitter vesicles.

### Possible indirect action of JhI-21 in adjusting synaptic strength at the larval NMJ

It has been previously shown that non-vesicular glutamate release is partially dependent on the amount of glutamate present in the motor neuron^6^. This intracellular glutamate pool is regulated via glutamate metabolizing enzymes. Glutamate oxaloacetate transaminase (GOT) produces glutamate from aspartate. Glutamate decarboxylase (GAD) catalyzes the decarboxylation of glutamate to γ-aminobutyric acid (GABA), and glutamate synthase (GS) converts glutamate to glutamine^4^,^44^. In addition glutamate dehydrogenase (GDH) catalyzes the reversible formation of glutamate to α-ketoglutarate^46^. It has been shown that misexpression of glutamate metabolizing enzymes alters postsynaptic iGluR clustering by regulating presynaptic intracellular glutamate concentrations^6^. Instead of directly transporting glutamate, JhI-21 could therefore function to transport metabolites, activators, or suppressors associated with regulation of glutamate levels.

For example, it has been shown that JhI-21 in S2 cells transports leucine, which acts as an allosteric activator of the glutamate dehydrogenase (Gdh) and could thereby modify intracellular glutamate levels (Fig. 10)^20^,^47^.

### Specific role of JhI-21 during late larval development

JhI-21 expression was also previously shown to be dependent on Juvenile Hormone (JH)^18^,^19^. The role of this hormone in many insects is to maintain juvenile morphological characteristics^48^. In *Drosophila*, several studies suggested a role for JH in adult behaviors like foraging and sexual maturation^19^,^49^. The role of JH in larvae, however, remains unclear. In *Drosophila* larvae, the titer of JH III, the active form of JH in Diptera, is high during the first and second larval stages. In feeding stage 3^rd^-instar larvae, JH III levels decrease drastically before increasing again during wandering prior to pupation^50^,^51^. These differences in JH III levels might be responsible for the differences of JhI-21 expression and subcellular localization that we observed in this study. This shift in JH III-dependent expression of JhI-21, and the resulting shift in postsynaptic sensitivity occurs at the same time as the behavioral switch in late 3^rd^-instar larvae. When placed on agar plate, which is low in nutrients, feeding larvae moved at a higher speed compared to wandering individuals. This hypermobility in feeding larvae could reflect aggressive searching for food. Wandering larvae, on the other hand, move at a lower pace but turn twice as much compared to feeding larvae (Fig. 7). This behavior could reflect a search for the best place to start pupation. Both behaviors are altered in *JhI-21* hypermorphs, where feeding and wandering animals move at nearly the same speed and display similar turning rates. Although we could demonstrate that JhI-21 is involved in the age dependent shift of synaptic strength between feeding and wandering larvae, we can not rule out that other JhI-21 expressing cells in the brain are presumably involved in those behavioral changes. In any case, it is nonetheless clear that JhI-21 is important for regulating the change of behavior during late 3^rd^-instar life.

In summary, we show that ERC values can be used to screen for proteins that work in the same neurotransmitter pathway especially when combined with a second screening method such as comparison of expression patterns. Using this method, we identified JhI-21 as a novel regulator of synaptic glutamate signaling. We found that JhI-21 is expressed in motor neurons where it regulates the developmental specificity of synaptic strength by inhibiting the clustering of post-synaptic iGluRs (Fig. 10). We were also able to highlight the role of JhI-21 on late larval locomotor activity, and show that changes in the subcellular distribution of JhI-21 within motor neurons might explain the differential regulation of JhI-21 on NMJ strength and behavior.

Glutamate is the predominant excitatory neurotransmitter in the central nervous system in mammals. Thus glutamate is involved in the control of a wide range of brain functions. It is also a key player in many neurological diseases^52^,^53^. In this context glutamate transporters play a key role in regulating extracellular glutamate levels to maintain dynamic synaptic signaling processes. Therefore our identification of JhI-21 as an important actor on glutamatergic and locomotor physiology in *Drosophila* is suggesting that its possible ortholog (LAT-1^36^) might also have an impact on such pathways in mammals. Beside the known glutamate transporters (e.g. vGluTs loading glutamate into synaptic vesicles, and EAATs removing the excess of glutamate by surrounding glial cells)^54^, other transporters such as LAT-1, which are possibly not transporting glutamate could also have a major impact on glutamate receptor physiology in the mammalian nervous system. This would be a major clue to explore new strategies to cure neurological diseases.

Our data are also highlighting the potential of a new emerging technique in finding genes that co-evolve: ERC. This technology proved to be extremely powerful in the recent past to find molecular partners that interact in the same cells^36^. Here we show that it has also the power to decipher specific pathways such as the glutamatergic physiology and the age specific control of locomotor activity even if the products of these genes are expressed in different cell types (JhI-21 and glutamate receptors). Therefore the ERC technique would be particularly useful to reveal some pluricellular molecular networks such as the interaction between glial cells and neurons, or the plasticity of interacting neurons during development or during a learning task by identifying genes that are co-evolving. Such applications are facilitated by the ERC analysis webserver that will perform custom analysis genome-wide for user-chosen genes^55^.

## Materials and Methods

### Calculation of evolutionary rate covariation statistics

ERC values were calculated as previously described^36^. Briefly, orthologous genes sequences from 12 *Drosophila* genomes were aligned and used to estimate gene-specific branch lengths across the species phylogeny. These branch lengths were transformed into relative rates of evolution based on the average genome-wide amount of divergence for each branch^10^. Rate covariation (ERC) values were calculated from the relative rates as a correlation coefficient for each gene pair. Statistical tests on groups of genes, such as the glutamate receptors, were performed by comparing the mean ERC value to 10,000 random sets of the same size using a permutation test.

### Drosophila stocks and genetics

Flies were grown and maintained on regular corn medium, at 25 °C, in a 12 h/12 h light/dark cycle. Control *Drosophila melanogaster* used in this study were *Oregon-R*, P*82 (precise excision of P{SUPor-P}*JhI-21*^*KG00977*^; electrophysiology and iGluR quantification), and *w*^*1118*^ (electrophysiology, iGluR quantification , and behavior). *JhI-21* mutants P{SUPor-P}*JhI-21*^*KG00977*^ (BL12970), P*JhI-21*^*EP1187*^ (not available any longer at Bloomington, but still available in our laboratory), and *Df(2L)esc-P3-0* (BL3131) were previously characterized^29^ and obtained from the Bloomington Stock Center. *JhI-21* alleles were re-balanced over *CyO-GFP*. P{*TubP- Gal4*}LL7*/TM3, Sb* was obtained from Bloomington (BL5138) and re-balanced over *TM3GFP*, *Ser*. UAS-*nSyb-GFP*^56^ provided by B. Hovemann, (Ruhr-University Bochum), *repo-Gal4* (BL7415, obtained from Bloominton), and 24-B-*Gal4* (BL1767, obtained from Bloomington), OK6-Gal4 and OK-371-Gal4 were provided by H. Abele (Heinrich-Heine University Düsseldorf). All genotypes used for behavioral experiments were backcrossed 5 times to an isogenized *w*^*1118*^ fly stock.

### JhI-21-Gal4

UAS-Sequences were removed from pUAST attB^57^ using EcoRI and HindIII. Vector was treated with Klenow fragment and religated to make attB. Heat-shock (HS) minimal promotor-, Gal4-, and hsp7αolyA-sequence were removed from pCHS-Gal4^58^ using NotI and cloned into attB to make Gal4 attB. JhI-21 promoter fragment was PCR amplified from genomic DNA of *Canton-S* flies by using primers, which introduced KpnI sites for cloning (underlined): GGTACCGGGATTCTTCTGCTTACCCTCT and GGTACCGCACCGATAGGAGGATGTATTC. The amplicon was subcloned to pGEM®-T Easy (Promega), re-excised and cloned to Gal4 attB using KpnI. The resulting transgenic flies were generated by site-directed integration using attB44 (provided by J. Bischof, University of Zürich). Injections were performed by Genetic Services Inc. (Sudbury, MA, USA).

### qPCR for quantification of JhI-21 expression

For reverse transcription RT-PCR, total RNA was isolated using Trizol (Invitrogen, Carlsbad, CA) extraction^59^. For the *JhI-21* expression experiments, total RNA was isolated from whole larvae. RNA (500 ng, quantified spectrophotometrically) was reverse transcribed using oligo-dT primers and standard methods. 10% of the cDNA product was used to amplify *JhI-21* and *actin5C* cDNA fragments by PCR. A 122 fragment of *JhI-21* was amplified using the following primer pair: TTGTTTACCACGGCGAAATAG and CTTTGTGACGGAGGAGCTACA; a 200 bp fragment of *actin5C* was simultaneously amplified using CAAGCCTCCATTCCCAAGAAC and CGTGAAATCGTCCGTGACATC primer pair.

Real-time PCR was performed using an MJResearch Opticon2 real-time thermocycler and quantitative fluorescent detection of SYBR green-labeled PCR product. Relative mRNA abundance was calculated using the “∆∆CT method”^60^.

### Immunocytochemistry and Confocal microscopy

2 Rabbit polyclonal anti-JhI-21 antibodies were raised against a synthesized peptide (DGEEKIVLKRKLTLINGVA) by Thermo Scientific/Open Biosystems, using standard methods and used at a dilution of 1:250. Both gave similar results, and we kept the one giving less background staining. The JhI-21 peptide epitope represents amino acids 30-48 of the predicted *Drosophila* JhI-21 protein. Specificity was verified on JhI-21 null mutant embryos (Figure S2).

NMJs were dissected under *Drosophila* saline supplied with 2 mM Glutamate. Glutamate present in the buffer prevents retraction of glia from the NMJ [4]. Imaging was performed on larval ventral longitudinal muscles (VLM) 6 and 7. For GluRIIA or GluRIIB stainings, NMJs were fixed 30 min in Bouin´s fixative (Sigma). Immunostaining measurements of postsynaptic glutamate receptor abundance were performed as previously described^4^,^6^,^31^,^33^,^61^. For JhI-21 localization experiments, NMJs and larval brains were dissected in PBS + 2 mM glutamate (NMJs) or directly in fixative (brains) followed by a 30 min fixation in 4% PFA in PBS. Primary antibodies were incubated overnight at 4 °C. Mouse monoclonal anti-GFP (Sigma Aldrich, Saint Quentin Fallavier, France) was used at 1:200. Mouse monoclonal anti-ElaV (9F8A9) was obtained from the University of Iowa Developmental Studies Hybridoma Bank (Iowa City, US) and used either at 1:200 or at 1:500. A 1-5 h washing step was performed with at least 3 solution changes, before the incubation of secondary antibodies for 2 h at RT (for embryos) or overnight at 4 °C (for the rest). DylightTM594-conjugated goat anti-Horseradish Peroxidase (HRP) antibody was obtained from Jackson ImmunoReserach Laboratories Inc. (West Grove, US) and used at 1:500. TRITC-conjugated anti-HRP antibody was obtained from Jackson ImmunoReserach Laboratories Inc. (West Grove, US) and used at 1:100. Secondary antibodies (Alexa Fluor 488 or 594 Dye) were obtained from Molecular Probes and were diluted at 1:400. Preparations were mounted in Vectashield (H-1000, Vector Labs) before imaging using a Leica TCS SP2 AOBS or an Olympus Fluoview FV500 laser scanning confocal system. Confocal projections were scanned at 1 μm section intervals, and were orientated and cropped with LeicaLight (also used to obtain Z-projections). Measurements of postsynaptic glutamate receptor density in confocal stacks were made by quantifying mean postsynaptic immunofluorescence intensity relative to fluorescence in surrounding muscle tissue: F synapse /F background membrane^4^.

### Electrophysiology

All electrophysiological recordings were obtained at 19 °C from third instar (110-120 hr after egg laying) larval ventral longitudinal muscle 6 (A3-A4) at -60 mV holding potential using two-electrode voltage clamp technique (TEVC), as previously described^4^,^62^,^63^. Dissections and electrophysiology were performed under glutamate free *Drosophila* HL-3 saline^4^. Electrodes for TEVC were filled with 3M KCl yielding a resistance of 30–40 MOhm. Spontaneous excitatory junctional currents (sEJCs) or “minis” were detected and analyzed using Clampfit9/10 template-matching method that identifies synaptic events based on shape matching to a data-based ideal template^33^. Axon GeneClamp 500B/AxoClamp 900A and Digidata 1550 (Molecular Devices, Sunnyvale, CA) were used for data acquisition.

The bathing experiment was performed with 2 mM glutamate during 24 h on NMJ cultures as previously described^4^.

### Staging of feeding and wandering LIII larvae

3^rd^-instar larvae were picked from the food (to obtain feeding stage larvae) or the wall (to obtain wandering stage larvae) of a stock vial and then transferred to a dish containing a piece of food for stage confirmation. Animals that stayed in the food were considered as “feeding” larvae. Larvae escaping the food were determined to be “wandering” 3^rd^-instar.

### Behavior

Staged larvae were washed in ddH_2_O before conducting behavioral analysis. Movement analysis was performed on a 9 cm-circular 2%-agar-agar medium. This agar medium was used upside down to have a perfect flat surface and was placed in the middle of a bigger Pétri dish (13 cm diameter). The space between the edge of the Pétri dish and the agar was filled with ddH_2_O to avoid escaping larvae. A single larva was placed on the middle of the agar. Recordings and analysis were performed automatically using EthoVision XT software (Noldus information technology, Wageningen, Netherlands). Dynamic subtraction function was chosen to distinguish the larva from the background. Data collection was started two minutes after the larva was placed on the agar plate. A ten-minute movie was recorded for each individual. Speed and meander behaviors were calculated using the same software, since these parameters reflect most of the locomotor activity characteristics of larvae in our behavioral set up.

### Lethality and Developmental time

Flies were allowed to lay eggs for 20 h. Approximately 3 × 100 embryos were transferred to fresh vials. 40 h after egg-laying, non-hatched embryos were counted. The amount of pupae was determined each day. After 10 days hatched adults were counted and the amount of lethality calculated.

### Statistics

All data were transferred to Prism 5.0d (Graphpad) for statistical analysis and tested for normal distribution using the D´Agostino and Pearson omnibus normality test. Two pairs of normally distributed data were analyzed using the paired-t-test. Pairs of data, which did not pass the normality test, were analyzed using the Mann-Whitney test. Three sets of not normally distributed data were analyzed by Kruskal-Wallis test; for quantification of GluRIIA and GluRIIB we compared both controls with each ‘test’ genotype (i.e. we did 3 comparisons for all 5 genotypes). Normally distributed data with two nominal variables were analyzed using the two-way ANOVA followed by Bonferroni post test. Survival curves were analyzed using the Chi2 test and cumulative frequency histograms by usage of the Kolmogorov-Smirnov test.

## Additional Information

**How to cite this article**: Ziegler, A. B. *et al.* The Amino Acid Transporter JhI-21 Coevolves with Glutamate Receptors, Impacts NMJ Physiology, and Influences Locomotor Activity in *Drosophila* Larvae. *Sci. Rep.* **6**, 19692; doi: 10.1038/srep19692 (2016).

## Supplementary Material

Supplementary Information

## Figures and Tables

**Table 1:** Glutamate receptors show overall positive ERC values and highest expression in the CNS or the body wall.

Gene	ERC Data		GluR expression pattern					
	ERC value	*p*-value	CNS	Body wall	DS	MT	Fat body	Trachea
mGluR	0.556	0.00021	**X**					
GluRIIA				**XX**	X	X		
GluRIIB			X	X	X	X		
GluRIIC				**XX**	X			
GluRIID				**XX**	X			
GluRIIE				**XX**	X			

The table shows the grouped ERC value of all six glutamate receptors together and its corresponding *p*-value. The positive ERC value indicates the strenght of evolutionary covariation between all 6 GluRs. The larval expression pattern of the glutamate receptors according to Flyatlas ( www.flybase.org) is summarized as followed: X = low expression (10 – 99), XX = moderate expression (100 – 499). Tissues are abrevated as followed: central nervous system (CNS), digestive system (DS), malpighian tubules (MT).

**Table 2:** Co-evolution of putative transporter genes with glutamate receptors and summary of their larval expression pattern.

Transporter	ERC Data		Transporter expression pattern					
	ERC value	*p*-value	CNS	Body wall	DS	MT	Fat body	Trachea
bdg	0.465	0.0001	X	X	X	X	X	X
CG8785	0.3828	0.0007			**XXXX**	XX		X
CG13384	0.4405	0.0007	XX	XX	XX	XX	**XXX**	XX
**JhI-21**	0.3788	0.0019	**XXX**	XX	XX	X	X	XX
CG16700	0.4073	0.0023	X	XX	XX	X		X
CG9413	0.305	0.0028	XX	XXX	XXXX	X		X
CG7255	0.4127	0.0045		X	X	X	X	
DAT	0.3223	0.0075	X					
mnd	0.2603	0.0122	XX	XX	X	XX	X	X
CG13795	0.23	0.0467		X	X	X	**XXXX**	
blot	0.183	0.0624	XX	XXX	XX	XX	X	XXX
ine	0.1678	0.0737	X	X	**XXXX**	XX		
gb	0.1493	0.1334	**XXX**	X	XX	X	XXX	X
CG1698	0.1077	0.2114		X	**XXX**	X		X
CG32079	0.107	0.2472						
VGlut	0.0648	0.2617	X					
CG43066	0.0193	0.4055						
SerT	0.0298	0.4108	X		X			
CG5535	0.0737	0.4433	XX	XX	XX	X	X	XX
CG4476	−0.0002	0.597	**XX**		X			
CG15279	−0.0185	0.6095		XX	X	**XXXX**		XX
CG13796	−0.0432	0.6126	X	XX	XX	X	**XXXX**	XX
CG4991	−0.063	0.7208		X	**XX**			X
CG12531	−0.072	0.600						
NAAT1	−0.0828	0.739			XX	XX		
Eaat1	−0.087	0.74	**XXX**		XX	X	XX	
path	−0.0598	0.797	**XXX**	XX	XX	X	XX	X
CG5549	−0.1158	0.8007						
slif	−0.1537	0.9087		XX	XXX	**XXXX**		X
CG7888	−0.1735	0.929	XX		X	**XXX**		
Vmat	−0.1715	0.9422	**XXX**		XX			
CG8850	−0.227	0.9604		X		XXX	XXX	
CG15088	−0.2275	0.9631	X		X	**XX**		
CG1139	−0.2303	0.9736		XXX	X	XXX		X
VGAT	−0.2725	0.9929	**X**					
CG1607	−0.3837	0.9988	**XXXX**	XX	XX	XX	XXX	XX
CG13248	−0.434	0.9998	**XX**		X			
CG10804	−0.435	0.9999	**XX**					
Eaat2	−0.5347	1.00	**XXX**					

The table shows group ERC values of 39 putative amino acid transporters and glutamate receptor subunits (mGluRA, GluRIIA, GluRIIB, GluRIIC, GluRIID, and GluRIIE) with their corresponding *p*-values. The magnitude of positive ERC values indicate the strength of evolutionary covariation between the amino acid transporter and the 6 GluRs. The expression pattern according to Fly Atlas ( www.flybase.org) is summarized as followed: X = low expression (10 – 99), XX = moderate expression (100 – 499), XXX = high level expression (500 – 999), XXXX = very high expression (>999). Tissues are abrevated as followed: central nervous system (CNS), digestive system (DS), malpighian tubules (MT).
