# Supplementary material for: The Amino Acid Transporter JhI-21 Coevolves with Glutamate Receptors, Impacts NMJ Physiology, and Influences Locomotor Activity in Drosophila Larvae
Source: Sci Rep. 2016 Jan 25;6:19692. doi: 10.1038/srep19692 (PMC4726445; doi:10.1038/srep19692)
Supplement: Supplementary Information [file srep19692-s1.pdf]

Supplementary information for: The Amino Acid Transporter JhI-21 Coevolves with Glutamate Receptors, Impacts NMJ Physiology, and Influences Locomotor Activity in *Drosophila* Larvae.

**Anna B. Ziegler[1,2,3], Hrvoje Augustin[4], Nathan L. Clark[5], Martine Berthelot-Grosjean[1,2,3] , Mégane M. Simonnet[1,2,3], Joern R. Steinert[6], Flore Geillon[1,2,3], Gérard Manière[1,2,3], David E. Featherstone[4] and Yael Grosjean[1,2,3*]**

[1]CNRS, UMR6265 Centre des Sciences du Goût et de l'Alimentation, F-21000 Dijon, France.
[2]INRA, UMR1324 Centre des Sciences du Goût et de l'Alimentation, F-21000 Dijon, France.
[3]Université de Bourgogne-Franche-Comté, UMR Centre des Sciences du Goût et de l'Alimentation, F-21000 Dijon, France.
[4]Biological Sciences, University of Illinois at Chicago, Illinois 60607, Chicago, USA
[5]Department of Computational and Systems Biology, University of Pittsburgh, Pittsburgh 15260, Pennsylvania, USA
[6]MRC Toxicology Unit, University of Leicester, LE1 9HN Leicester, UK

H.A.´s present address: Institute of Healthy Ageing, and GEE, University College London, WC1E 6BT London, UK

* Corresponding author:
e-mail address: yael.grosjean@u-bourgogne.fr

**Supplement Materials and Methods:**

*FLP-FRT-based JhI-21 deletion*

The DrosDel isogenic deficiency kit was used to design a null allele for *JhI-21*[1]. Transposons containing FRT compatible sites and covering *JhI-21* gene were selected using the following web site: http://www.drosdel.org.uk/fdd/del_hunter.php

Because of several misannotations of transposon insertions in this website, the smallest deletion we were able to generate, which could lead accurately to a null allele, was obtained using the following strains:

-$w^{1118;}$ P{RS5}*CG6770*[5-HA-5095], inserted at 12045.952kb (DGRC#125824)

-$w^{1118}$; PBac{RB}e00136, inserted at 12063.132kb (Exelixis stock e00136).

A two-step cross scheme was carried out:

Step 1: *w*/y; P{RS5}*CG6770*[5-HA-5095]/P{RS5}*CG6770*[5-HA-5095]; +/+ males were crossed to *w*,hs*FLP*/*w*,hs*FLP*; *Sp*/*CyO*; *Dr*/*TM6B* females (Bloomington-BL8862, modified with markers on $II^d$ and $III^d$ chromosomes).

Step 2: *w*,hs*FLP*/y; P{RS5}*CG6770*[5-HA-5095]/*CyO; +*/*TM6B* resulting males were mated to *w*/*w*; PBac{RB}e00136/*CyO*; +/+ females.

After 72 hours, parents and progeny (including *w*,hs*FLP*/*w*; P{RS5}*CG6770*[5-HA-5095]/ *PBac{RB}e00136*; +/+) of this second cross were placed at 37 °C for 1hr to start the expression of the Flippase (FLP) necessary to rearrange the chromosome and to eliminate *JhI-21*. Parents were then removed. The following day, eggs and larvae were placed again at 37 °C for 1hr. This heat shock was repeated once a day for 4 days. Emerging adult females were collected and balanced over *CyOGFP*.

13 *JhI-21* deletion candidates were screened by PCR using the following primers: CCCAAATTTGTGATACCCACTT (in P{RS5}*CG6770*[5-HA-5095]) and ATGCTTGGATTTCACTGGAACT (in PBac{RB}e00136). These primers could amplify a 699bp fragment if the deletion was successful. The resulting deletion span 17.2kb and

removed entirely 5 genes (*CG6792*, *CG14945*, *JhI-21*, *CG34164*, and *CG5317*) and partially 2 neighboring genes (*CG6770* and *rab3-GAP*). 2 successful candidates were obtained.

*Anti-JhI-21 labeling of embryos:*

Flies were allowed to lay eggs for three hours. Embryos were collected at 13-16 h after egg laying, briefly rinsed with water and dechorionized with 4 % bleach for 2-3 minutes, and rinsed with embryo wash solution (7 % NaCl, 0.5 % Triton-X-100). Embryos were transferred to heptane and fixed by adding an equal volume of 4 % PFA/PBS solution. The PFA and Heptane solutions were removed after 20 minutes and replaced by Methanol. Embryos were rehydrated for 15 min using PBST (PBS, 0.1 % TritonX-100) and blocked for 30 min in PBST + 5 % NGS. Embryos were incubated with anti-JhI-21 (1:250) and mouse anti-GFP (1:100) in 1 % NGS for 2 h and washed six times 10 min using PBST. Embryos were blocked for 1 h and incubated with secondary antibodies for 2 h. After 6 washes of 10 minutes embryos were mount in Vectashield 1000.

NMJ electrophysiology for eEJCs

Electrophysiological recordings were obtained at 19 °C from third instar (110-120 hr after egg laying) larval ventral longitudinal muscle 6 (A3-A4) at -60 mV holding potential using two-electrode voltage clamp technique (TEVC). Dissections and electrophysiology were performed under glutamate free *Drosophila* HL-3 saline. Electrodes for TEVC were filled with 3M KCl yielding a resistance of 30-40 MOhm. Synaptic current were evoked at 1 mM extracellular $Ca^{2+}$ and average single eEJC amplitudes (stimulus: 0.1ms, 1-5V) are based on the mean peak eEJC amplitude in response to ten presynaptic stimuli (recorded at 0.2Hz). Nerve stimulation was performed with an isolated stimulator (DS2A, Digitimer). All data were digitized at 10kHz.

*Other locomotor parameters:*

Perti dishes were filled with 2 % agar-agar in $ddH_2O$. Larvae of the appropriate genotype and stage were placed on the agar-agar surface and allowed to adapt to their new environment for 30 seconds. Larvae were observed using a bionocular (Brand) and peristaltic waves as well as the number of stops were manually counted for 2 minutes.

*Glutamate transport:*

Drosophila Schneider 2 cells (Invitrogen) were cultivated at 25°C in Schneider's medium, with 10% (v/v) of heat inactivated fetal calf serum. Uptake experiments were realized in 24 well plates, when cells reached 80 % confluence. Cells were washed 3 times with sodium-free buffer (choline chloride 125.0 mM, KCl 4.8 mM, CaCl2 1.3 mM, MgSO4 1.2 mM, HEPES 25.0 mM, KH2PO4 1.2 mM, Glucose 5.6 mM, pH7.4) and incubated 15 minutes in the same buffer. Glutamate uptake into Schneider 2 cells was realized for 5 minutes at room temperature (25 °C) and at 4°C, using concentrations varying from 50 µm to 500 µm and different ratios between non-radiolabeled glutamate and radiolabeled glutamate (L-[3,4-3H]-glutamic acid; 1 mCi.mL-1; 47.5 Ci.mmol-1; Perkin Elmer) (1:250 – 1:5000), added in 500 µL of sodium-free buffer. The uptake was stopped by washing cells 3 times with cold sodium-free buffer in which 20 mM glutamate was added to avoid release of previously up-taken glutamate. Cell lysis was obtained by adding 1 mL of lysis buffer (NaOH 0.1%; SDS 0.1%). 5 ml of scintillation medium (Ultima Gold XR, Perkin Elmer) were added to 500 µL of lysate and dpi per minute were counted by a scintillation counter for 3 minutes (Tri-Carb 2900 TR, Perkin Elmer).

As a positive control for amino acid net uptake leucine at a concentration of 50 µm and at a ratio of 1:2000 between cold and radiolabeled leucine (L-[3,4,5-3H(N)]-Leucine; 5 mCi.mL-1; 112 Ci.mmol-1; Perkin Elmer) was used. Uptake experiments followed the same

protocol, except that leucine uptake was measured for 1 min and 20 mM of leucine were added in sodium-free buffer to wash cells before lysis.

For the inhibition experiments, the uptake of 20 µM Leucine (with a ratio of 1:1500 between cold and radiolabeled Leucine) was measured in the presence or absence of 2 or 10 mM nonlabeled test amino acids. Uptake experiments followed the same protocol as described before for leucine uptake. Experiments were realized at 25° and at 4°C for the background and final results represent net leucine uptake.

**Supplement Reference**

1. Ryder, E., et al., *The DrosDel deletion collection: a Drosophila genomewide chromosomal deficiency resource.* Genetics, 2007. **177**(1): p. 615-29.

**Supplement Figures:**

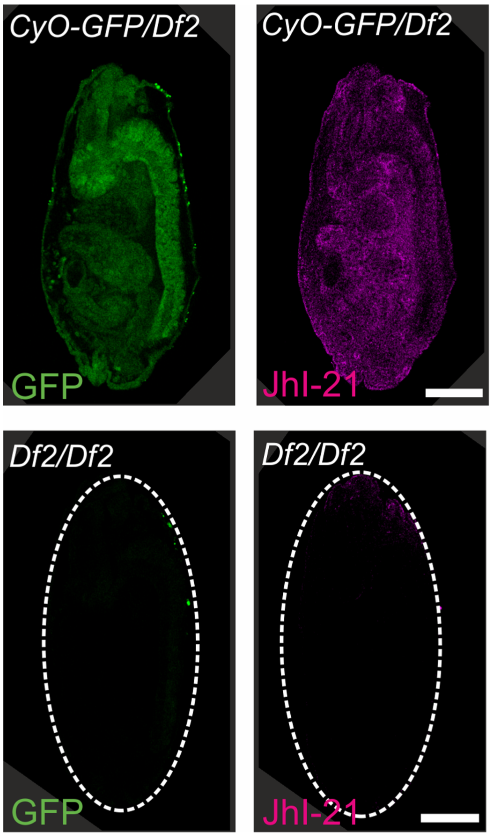


**Figure S1. Specificity of anti-JhI-21.** Since no null mutant was available to test the antibody specificity, we generated the smallest possible deficiency using the DrosDel kit [60]. *JhI-21*, as well as 6 other genes, have been removed totally or partially in the resulting deficiency allele *Df2*. Because *Df2*/*Df2* individuals die during 1$^{st}$-instar larval stage, we used homozygous *Df2*/*Df2* embryos to test the antibody specificity. Maternal *JhI-21* mRNA has been shown to be present in high amount (0-2 hours after egg laying) and then decrease dramatically six hours after egg laying [19]. Therefore, we measured anti-JhI-21 labeling in late embryos (about 18 hours after egg laying). Confocal images of late stage *Drosophila* embryos stained with antibodies against JhI-21 (magenta) and GFP (green). Heterozygote *JhI-21* mutants (*CyO-GFP*/*Df2*) are labeled by GFP expression and show distinct JhI-21 labeling, which is strongest present in the central nervous system (CNS). Embryos homozygote mutant for *JhI-21* (*Df2*/*Df2*) do not reveal any anti-JhI-21 labeling. Scale Bar = 80 mm.

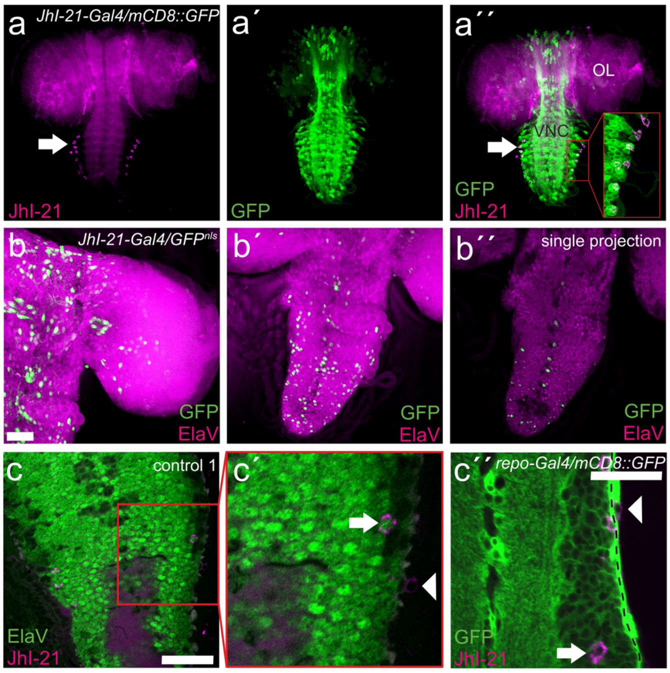


**Figure S2. Expression of JhI-21 in the larval brain**. JhI-21 is expressed in neurons in the central nervous system of 3rd-instar larvae. **a,** Confocal projection of a larval brain stained with antibodies to JhI-21 (magenta) and GFP (green). Anti-JhI-21 labels the neuropile of larval brains and few cell bodies at the ventral nerve cord (VNC). Anti JhI-21 labeling co-localizes with *JhI-21*-Gal4 driven mCD8-GFP expression (arrow). **b,** Confocal projection of a larval brain stained with antibodies against *JhI-21*-Gal4 driven UAS-GFP$^{nls}$ (green) and neuronal marker ElaV (magenta). Within the larval brain *JhI-21*-Gal4 is expressed only in neurons. **c,** Confocal Image of the VNC stained with antibodies to JhI-21 (magenta) and ElaV (green). Cells at the VNC labeled by anti-JhI-21 are marked by neuronal marker anti-ElaV (arrow). The arrowhead points at one JhI-21 positive cell, which is not marked by ElaV expression. **d,** Confocal image of the VNC stained with antibodies to JhI-21 (magenta) and GFP (green). Anti-JhI-21 labeled cells at the VNC do not co-stain with repo-Gal4 driven mCD8-GFP (arrow and arrowhead). Anti JhI-21 positive cell (arrowhead) is localized peripheral to the blood brain barrier (BBB) indicated by strong expression of *repo*-Gal4 driven mCD8-GFP (striped line). Scale Bar = 40 mm.

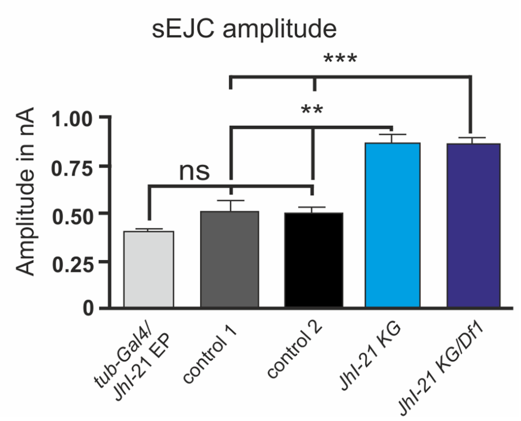


**Figure S3. Quantification of mean amplitudes of spontaneous excitatory junctional currents (sEJCs) from different genotypes in third-instar wandering *Drosophila* larvae.** Data were compared using a Kruskal-Wallis test between controls and mutants. ns: not significant; **: p<0.01 ***: p<0.001. N = 7-10 animals, 800-3,400 events measured per phenotype.

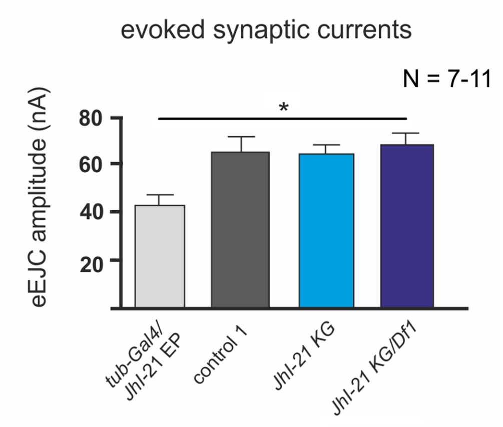


**Figure S4. Evoked synaptic currents are unaffected by Jhl-21 activity.** eEJCs were measured in TECV (at 1mM $Ca^{2+}$) for control 1 allele ($w^{1118}$), JhI-21 overexpression (*tub-Gal4/JhI-21* EP) and hypomorphic (*JhI-21 KG* and *JhI-21 KG/Df1*) alleles. Data were compared using a Kruskal-Wallis test (which gives a significant difference between the 4 genotypes, suggesting that the overexpression is different from the others: *: P = 0.0391 as indicated on the graph), but a Dunn's *post hoc* test gave no statistical difference between the

genotypes compared 2 by 2. Number of recordings (N) from at least 3 different larvae per genotype is indicated on the figure.

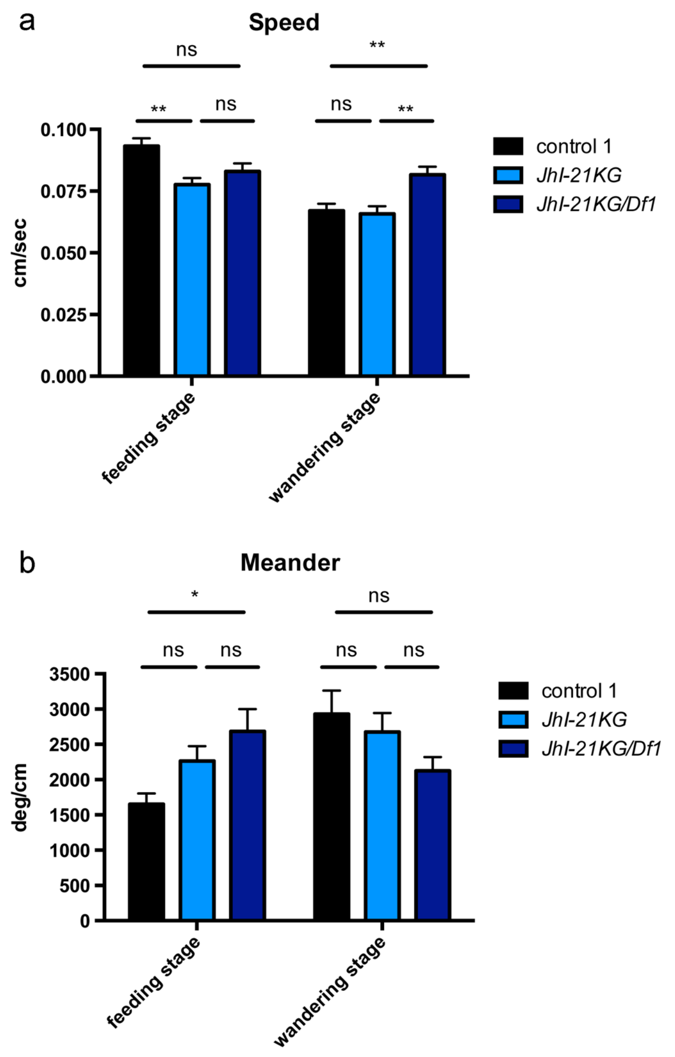


**Figure S5. Comparison between genotypes for locomotor behaviors. a-b** Parameters of locomotion were analyzed in wildtype larvae (control 1, $w^{1118}$) and *JhI-21* mutants (*JhI-21 KG* and *JhI-21 KG*/*Df1*). The data are identical to the data shown in Figure 7a and b, but comparing differences between genotypes instead of stages. Data were compared using a 2way ANOVA test followed by a Bonferroni *post hoc* test. **: $p < 0.01$; *: $p < 0.05$; ns: not significant. N=30 for each genotypes.

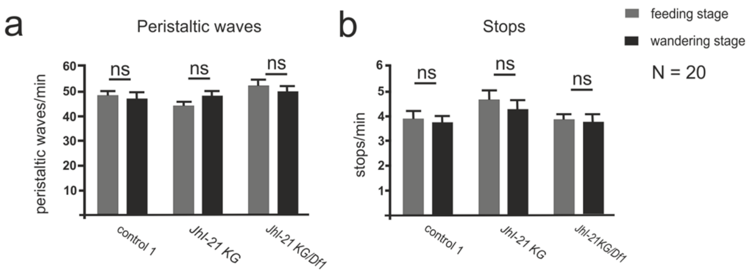


**Figure S6. Other locomotor parameters. a**, number of peristaltic waves along the larval body per min. **b**, number of larval stops per min. (N=20 for all genotypes and stages). Data were compared using a 2way ANOVA test followed by a Bonferroni multiple comparison *post hoc* test. ns: not significant.

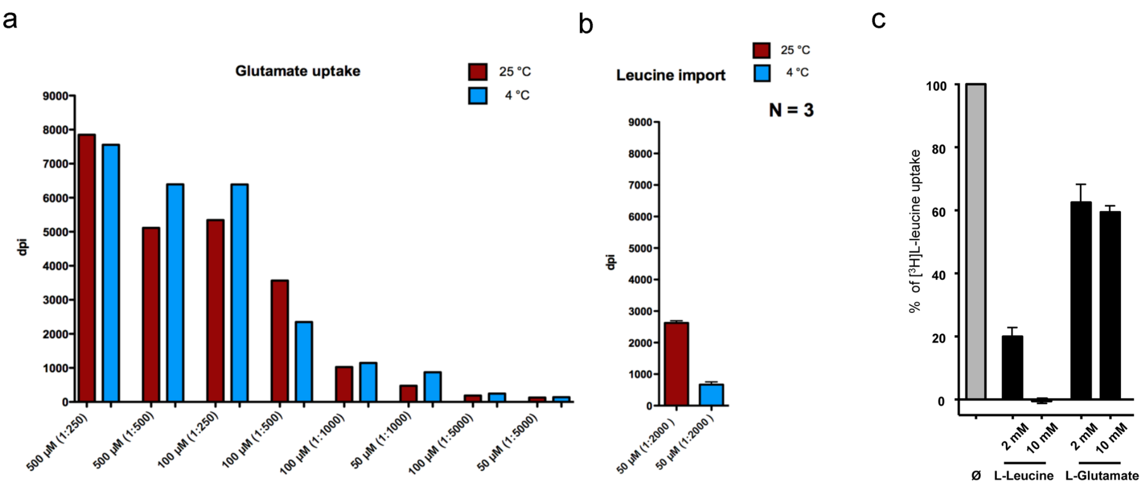


**Figure S7. Glutamate and leucine uptake into Drosophila S2 cells. a**, Different concentrations of glutamate and various ratio of cold/radiolabeled glutamate were used to test the possibility to uptake this amino acid into S2 cells at 25 °C within 5 minutes. Each value is compared to the background signal at 4 °C corresponding to glutamate unspecifically bound to the cell surface. Since glutamate uptake between 25 °C and 4 °C does not differ in any tested condition, we conclude that glutamate is not imported into S2 cells and hence the impact of JhI-21 on glutamate translocation cannot be proven. **b**, As a positive control we tested if radiolabelled leucine could be loaded in S2 cells [20]. Since the values for leucine uptake are drastically higher at 25 °C than at 4 °C, we conclude that in contrast to glutamate

leucine can be taken up to S2 cells. **c**, Inhibition of [3H]L-leucine by L-amino acids in S2 cells. The [3H]L-leucine uptake was measured in the presence of 2 or 10 mM non radiolabeled amino acids in the Na+-free uptake solution. Uptake experiments were realized at 25°C and at 4°C, and final results correspond to net uptake of leucine (N=3 for c).

**Table S1. ERC Values of putative amino acid transporters and glutamate receptors.**

| Gene name | mGluRA | | GluRIIA | | GluRIIB | | GluRIIC | | GluRD | | GluRIIE | |
|---|---|---|---|---|---|---|---|---|---|---|---|---|
| | ERC value | *p-value* | ERC value | *p-value* | ERC value | *p-value* | ERC value | *p-value* | ERC value | *p-value* | ERC value | *p-value* |
| bdg | 0.41 | 0.12365 | 0.513 | 0.06785 | 0.26 | 0.2384 | 0.708 | 0.0156 | 0.462 | 0.0873 | 0.437 | 0.11355 |
| CG8785 | 0.119 | 0.365 | 0.548 | 0.0409 | 0.095 | 0.388 | 0.48 | 0.08025 | 0.471 | 0.07055 | 0.584 | 0.034 |
| CG13384 | 0.582 | 0.04605 | 0.485 | 0.08335 | 0.273 | 0.22885 | 0.671 | 0.02405 | 0.272 | 0.22455 | 0.36 | 0.16905 |
| **JhI-21** | **0.59** | **0.0391** | **0.298** | **0.2058** | **0.309** | **0.1971** | **0.408** | **0.1336** | **0.304** | **0.1949** | **0.364** | **0.1635** |
| CG16700 | 0.663 | 0.0245 | 0.332 | 0.17915 | 0.816 | 0.00465 | 0.527 | 0.0777 | 0.086 | 0.38325 | 0.02 | 0.4564 |
| CG9413 | 0.567 | 0.035 | 0.197 | 0.2702 | 0.484 | 0.0628 | 0.333 | 0.1563 | 0.024 | 0.461 | 0.225 | 0.2433 |
| CG7255 | 0.579 | 0.062 | 0.457 | 0.1163 | 0.08 | 0.4076 | 0.69 | 0.027 | 0.235 | 0.27 | 0.435 | 0.13675 |
| DAT | 0.491 | 0.0792 | 0.548 | 0.0486 | 0.103 | 0.3725 | 0.138 | 0.3579 | 0.356 | 0.1496 | 0.298 | 0.2076 |
| mnd | 0.09 | 0.3796 | 0.404 | 0.1064 | 0.238 | 0.2303 | 0.145 | 0.3343 | 0.462 | 0.0698 | 0.223 | 0.2514 |
| CG13795 | 0.297 | 0.2039 | 0.22 | 0.2785 | 0.602 | 0.0351 | 0.139 | 0.3656 | 0.081 | 0.4133 | 0.041 | 0.4615 |
| blot | 0.393 | 0.1089 | 0.193 | 0.2796 | 0.124 | 0.3435 | 0.208 | 0.2702 | -0.058 | 0.5953 | 0.238 | 0.2349 |
| ine | 0.082 | 0.39275 | 0.397 | 0.1043 | -0.001 | 0.5073 | 0.006 | 0.5045 | 0.255 | 0.2081 | 0.268 | 0.2087 |
| gb | 0.46 | 0.0856 | -0.149 | 0.6911 | 0.42 | 0.1079 | 0.138 | 0.3597 | -0.096 | 0.6293 | 0.123 | 0.3696 |
| CG1698 | 0.178 | 0.29915 | -0.015 | 0.53025 | 0.472 | 0.0837 | 0.071 | 0.42675 | 0.017 | 0.47465 | -0.077 | 0.59545 |
| CG32079 | 0.31 | 0.2072 | -0.225 | 0.7353 | 0.688 | 0.0246 | 0.194 | 0.3173 | -0.158 | 0.6687 | -0.167 | 0.6687 |
| VGlut | 0 | 0.4379 | 0.082 | 0.36485 | 0 | 0.4351 | 0.131 | 0.32115 | 0 | 0.44995 | 0.176 | 0.2716 |
| CG43066 | 0.058 | 0.3997 | -0.016 | 0.5171 | 0.19 | 0.2439 | -0.117 | 0.6611 | -0.092 | 0.6239 | 0.093 | 0.3664 |
| SerT | 0.233 | 0.2473 | -0.255 | 0.7866 | 0.287 | 0.2025 | 0.092 | 0.395 | -0.094 | 0.62 | -0.084 | 0.6057 |
| CG5535 | -0.036 | 0.5529 | 0.244 | 0.3128 | -0.628 | 0.9428 | 0.23 | 0.3327 | 0.15 | 0.3811 | 0.482 | 0.1668 |
| CG4476 | -0.236 | 0.7251 | 0.145 | 0.3928 | -0.741 | 0.9754 | 0.019 | 0.5059 | 0.254 | 0.2996 | 0.558 | 0.1304 |
| CG15279 | 0.243 | 0.2284 | 0.044 | 0.4505 | -0.023 | 0.534 | 0.045 | 0.4573 | -0.324 | 0.8706 | -0.096 | 0.6277 |
| CG13796 | 0.202 | 0.2486 | -0.125 | 0.6585 | 0.207 | 0.2436 | -0.139 | 0.6732 | -0.124 | 0.6544 | -0.28 | 0.8111 |
| CG4991 | 0 | 0.4659 | -0.168 | 0.7151 | 0 | 0.4631 | -0.104 | 0.6458 | 0 | 0.4779 | -0.106 | 0.6428 |
| CG12531 | -0.028 | 0.4928 | -0.107 | 0.6259 | -0.153 | 0.6991 | -0.175 | 0.7316 | -0.085 | 0.5859 | 0.118 | 0.2797 |
| NAAT1 | 0 | 0.4686 | -0.297 | 0.8248 | -0.028 | 0.5299 | 0.079 | 0.4079 | -0.163 | 0.6866 | -0.088 | 0.5991 |
| Eaat1 | 0.251 | 0.2188 | -0.142 | 0.6658 | 0.124 | 0.3325 | -0.387 | 0.8842 | -0.139 | 0.6606 | -0.229 | 0.7482 |
| path | -0.252 | 0.8343 | 0.099 | 0.3712 | 0.109 | 0.3449 | 0.087 | 0.3944 | -0.171 | 0.7504 | -0.231 | 0.8034 |
| CG5549 | 0.042 | 0.4334 | -0.089 | 0.6084 | -0.005 | 0.5045 | -0.411 | 0.8855 | 0.035 | 0.4507 | -0.267 | 0.78 |
| slif | -0.499 | 0.953 | -0.049 | 0.575 | -0.32 | 0.8505 | 0.078 | 0.4183 | -0.071 | 0.5988 | -0.061 | 0.5841 |
| CG7888 | -0.082 | 0.6076 | -0.159 | 0.7001 | -0.453 | 0.9392 | -0.314 | 0.839 | -0.097 | 0.626 | 0.064 | 0.4215 |
| Vmat | -0.226 | 0.7708 | -0.072 | 0.5915 | -0.206 | 0.7479 | -0.141 | 0.6742 | -0.319 | 0.8614 | -0.065 | 0.5787 |
| CG8850 | 0.028 | 0.437 | -0.342 | 0.8679 | 0.053 | 0.4065 | -0.249 | 0.7705 | -0.523 | 0.966 | -0.329 | 0.8374 |
| CG15088 | -0.164 | 0.6884 | -0.236 | 0.76695 | 0.077 | 0.3861 | -0.221 | 0.74215 | -0.318 | 0.8421 | -0.503 | 0.94225 |
| CG1139 | -0.332 | 0.8579 | -0.233 | 0.7677 | -0.306 | 0.8379 | -0.23 | 0.7551 | -0.261 | 0.795 | -0.02 | 0.5075 |
| VGAT | -0.275 | 0.82065 | -0.258 | 0.80895 | -0.22 | 0.7642 | -0.16 | 0.69365 | -0.43 | 0.9309 | -0.292 | 0.82485 |
| CG1607 | -0.396 | 0.8961 | -0.32 | 0.8467 | -0.129 | 0.65065 | -0.488 | 0.93705 | -0.531 | 0.9652 | -0.438 | 0.9099 |
| CG13248 | -0.207 | 0.73935 | -0.348 | 0.87725 | -0.408 | 0.9133 | -0.565 | 0.96515 | -0.466 | 0.93995 | -0.61 | 0.9769 |
| CG10804 | -0.588 | 0.9774 | -0.324 | 0.8443 | -0.462 | 0.9343 | -0.594 | 0.972 | -0.375 | 0.8794 | -0.267 | 0.7755 |
| Eaat2 | -0.472 | 0.9346 | -0.391 | 0.8976 | -0.53 | 0.9628 | -0.741 | 0.99505 | -0.532 | 0.9612 | -0.542 | 0.9551 |
